# Comparison of two recombinant erythropoietin formulations in patients with anemia due to end-stage renal disease on hemodialysis: A parallel, randomized, double blind study

**DOI:** 10.1186/1471-2369-6-5

**Published:** 2005-05-23

**Authors:** Jorge F Pérez-Oliva, Martha Casanova-González, Idrian García-García, Pedro J Porrero-Martín, Carmen M Valenzuela-Silva, Tairí Hernández-Montero, Marcia Lagarde-Ampudia, Yuri Casanova-Kutsareva, Yisel Ávila-Albuerne, Alicia Vargas-Batista, Hailen Bobillo-López, Raúl Herrera-Valdés, Pedro A López-Saura

**Affiliations:** 1 Hemodialysis Department, National Institute of Nephrology, Havana, Cuba; 2 Nephrology Department, "Gustavo Aldereguía Lima" Hospital, Cienfuegos, Cuba; 3 Clinical Trials Division, Center for Biological Research, Havana, Cuba; 4 Clinical Laboratory Department, National Institute of Nephrology, Havana, Cuba; 5 Clinical Trials Department, National Center of Clinical Trials, Havana, Cuba

## Abstract

**Background:**

Recombinant human erythropoietin (EPO) is used for the treatment of last stage renal anemia. A new EPO preparation was obtained in Cuba in order to make this treatment fully nationally available. The aim of this study was to compare the pharmacokinetic, pharmacodynamic and safety properties of two recombinant EPO formulations in patients with anemia due to end-stage renal disease on hemodialysis.

**Methods:**

A parallel, randomized, double blind study was performed. A single 100 IU/Kg EPO dose was administered subcutaneously. Heberitro (Heber Biotec, Havana, formulation A), a newly developed product and Eprex (CILAG AG, Switzerland, formulation B), as reference treatment were compared. Thirty-four patients with anemia due to end-stage renal disease on hemodialysis were included. Patients had not received EPO previously. Serum EPO level was measured by enzyme immunoassay (EIA) during 120 hours after administration. Clinical and laboratory variables were determined as pharmacodynamic and safety criteria until 216 hours.

**Results:**

Both groups of patients were similar regarding all demographic and baseline characteristics. EPO kinetics profiles were similar for both formulations; the pharmacokinetic parameters were very close (i.e., AUC: 4667 vs. 4918 mIU.h/mL; Cmax: 119.1 vs. 119.7 mIU/mL; Tmax: 13.9 vs. 18.1 h; half-life, 20.0 vs. 22.5 h for formulations A and B, respectively). The 90% confidence intervals for the ratio between both products regarding these metrics were close to the 0.8 – 1.25 range, considered necessary for bioequivalence. Differences did not reach 20% in any case and were not determined by a formulation effect, but probably by a patients' variability effect. Concerning pharmacodynamic features, a high similitude in reticulocyte counts increments until 216 hours and the percentage decrease in serum iron until 120 hours was observed. There were no differences between formulations regarding the adverse events and their intensity. The more frequent events were pain at injection site (35.3%) and hypertension (29%). Additionally, further treatment of the patients with the study product yielded satisfactory increases in hemoglobin and hematocrit values.

**Conclusion:**

The formulations are comparable. The newly developed product should be acceptable for long-term application.

## Background

Erythropoietin (EPO) is a sialoglycoprotein hormone secreted primarily by the mature kidneys in response to tissue hypoxia and/or red cell mass decrease. It stimulates erythrocyte production from the blood marrow [1]. Recombinant human EPO has been used since the 80's in the treatment of anemia associated to end-stage renal disease. Reports in hemodialysis patients indicate an effective erythropoiesis increment, ceasing or decrease of transfusion frequency, and quality of life improvement [2-6]. However, this treatment is still expensive, not affordable to developing countries if an extensive use, for all patients who need it, is sought, considering the same prevalence as in the United States, where there are currently 270 000 patients in dialysis [7,8].

Whether multiorigin recombinant proteins can be clinically interchangeable despite coming from different strains and manufacturing processes is a controversial matter nowadays [9,10]. If this principle is accepted, then a study that shows pharmacological similarity between two products would be enough to indicate its therapeutic interchangeability, given that they are chemically and pharmaceutically equivalent as well.

Recently, a recombinant EPO formulation was obtained in mammalian cells at the Center for Genetic Engineering and Biotechnology (CIGB in Spanish), Havana. Its pharmacokinetic, pharmacodynamic and safety profiles were studied, compared to a similar, previously existing preparation. The reticulocyte count increments and serum iron consumption were used as biological action indicators. Hemoglobin and hematocrit increases after 3-month treatment was used as an additional efficacy criterion as well.

## Methods

### Subjects

Patients, 18–75 years, both sexes, with anemia (hematocrit ≤ 28% or hemoglobin ≤ 9 g/dL) due to end-stage renal disease on hemodialysis (3 sessions per week), who gave their written, informed consent to participate were included. The exclusion criteria were: previous EPO treatment; pregnancy or nursing; sepsis or active infection; non-treated iron deficiency (serum iron <60 mg/dL, ferritin <100 ng/mL, transferrin saturation index <20%); cancer; hormonal treatment (except thyroid hormone, contraceptive and insulin); liver disease (twice and half the transaminases normal values); diastolic arterial tension ≥ 120 and/or systolic ≥ 180 mmHg; severe psychiatric dysfunction or another limitation that prevented the patient to give his consent; epilepsy; thrombocytosis (≥ 500 000 /mm^3^); signs of active bleeding; bone marrow aplasia; active collagen disease; severe hyperparathyroidism (serum parathormone > 400 ng/L), and hyperaluminemia (serum aluminum > 50 ng/mL). They were withdrawn from the trial if they died, abandoned voluntarily, had severe adverse reactions, were subjected to renal transplant, used not allowed medications or other EPO formulations, or if any exclusion criteria arose. The trial was in compliance with the Helsinki declaration. The protocol was approved by the Ethics Committee of the participant institutions and by the Cuban Regulatory Authority.

### EPO formulations

Formulation A (Heberitro, Heber Biotec, Havana) was in vials containing 2000 IU of human recombinant (rHu) EPO (produced in Chinese hamster ovarian (CHO) cells at CIGB, Havana), 2.5 mg serum human albumin, 0.2 mg polysorbate 20, 2.9 mg NaCl, 4.6 mg NaH_2_PO_4_.2H_2_O, 9.9 mg Na_2_HPO_4_, and water for injection to complete 1 mL. Formulation B consisted in a commercially available preparation (Eprex, CILAG AG, Switzerland) presented in pre-filled syringes containing 2000 IU rHuEPO, 0.15 mg polysorbate 80, 2.192 mg NaCl, 0.580 mg NaH2PO_4_.2H_2_O, 1.115 mg Na_2_HPO_4_.2H_2_O, 2.50 mg glycine, and water for injection to complete 0.5 mL.

### Study design

Subjects were distributed according to a computer-generated random number list, stratified by study center, to receive subcutaneously a single dose of 100 IU/Kg of one of the EPO preparations (A or B) in a parallel design. The study was double blinded. As the presentation of the products differed, blinding was kept by loading formulation A vials in syringes coded with the patients' inclusion numbers, and the nurse that administered the products did not participate in the rest of the trial.

Patients were hospitalized throughout the study under strict medical supervision. EPO administrations and blood sampling were done post-hemodyalisis, when renal chronic failure patients are closer to their ideal dry weight. Antipyretic medication was given orally if flu-like symptoms or pain at the injection site arose. If blood hypertension occurred during follow-up patients were treated according to the investigator's criteria and the precedent individual treatments.

After the 9 days in-hospital follow-up for the pharmacological comparison, both groups of patients continued treatment with formulation A, 30 UI per Kg 3 times per week, subcutaneously, in order to obtain additional efficacy and safety data for this product. Injections were administered after the hemodialysis sessions at the patients' current treatment centers.

### Clinical and laboratory evaluations

Blood samples for serum EPO concentration determinations were collected by venipuncture, before and 2, 6, 10, 14, 17, 20, 24, 48, 72, 96, and 120 hours after injection. Pharmacodynamics was assessed by reticulocyte counts before and at 48, 72, 120, 144 and 216 hours, and also by serum iron consumption, before and at 120 and 216 hours. Other hematological determinations (hemoglobin, hematocrit, red blood cell, platelet, and total and differential leukocyte counts) were taken as safety variables, every 24 hours. Alanine aminotransferase (ALT) was measured before and at the end of the in-patient period (216 hours). Patients were regularly checked for vital signs and symptoms, including during the hemodialysis sessions.

EPO was quantified in serum with a highly sensitive enzyme immunoassay (EIA) kit (Quantikine^® ^IVD^®^, R&D System, Inc, Minneapolis). Serum iron concentrations were determined by a colorimetric test (NITRO-PAPS, CENTIS, Havana). Blood chemistry and hematological counts were done according to usual clinical laboratory procedures. All laboratory analyses were done blindly.

Anti-EPO antibodies were screened at the end of the further treatment and follow-up period by a semiquantitative, "sandwich type", EIA system (EPO; sample; protein A – horseradish peroxidase conjugate) developed and validated at the Center for Biological Research, Clinical Laboratory (manuscript in preparation). For this purpose the blood sample was taken 72 hours after the last EPO administration. If some sample resulted positive it was previewed that the patient's baseline sample would be tested in order to discard spontaneous pre-existing autoantibodies.

### Data analysis

The drug disposition data analysis was performed per individual by a non-compartmental method with a combined linear/log – linear trapezoidal rule approach. The linear trapezoidal rule was used up to peak level and the logarithmic trapezoidal rule thereafter. The first-order rate constant associated with the curve terminal (log linear) portion (λ) and elimination half-life (t_1/2_) were estimated by linear regression of the included terminal data points. Time-to-peak values (Tmax) were determined directly from the experimental data as the time of maximum observed level (Cmax) considering the entire curve. Area under the serum concentration-time curve from 0 to 120 hours (AUC_120_) was calculated using the linear/log linear trapezoidal rule. Mean residence time (MRT) was also calculated using the moments of the drug disposition curve. Parameters that were extrapolated to infinity, such as AUC (area under disposition curve) and AUMC (area under first moment of the disposition curve), were computed based on the last predicted value from the linear regression performed to estimate λ and t_1/2_. In addition, other pharmacokinetic parameters were also calculated, such as the systemic clearance (CL), volume of distribution (Vd), and the peak to area ratio value (CAV = Cmax / AUC). The apparent absolute bioavailability (F) was estimated using data from a previously reported study after intravenous dosing of recombinant EPO as the subcutaneous to intravenous AUC ratio [11]. The WinNonlin professional software (Version 2.1, Pharsight Inc., 1997, NC, USA) was used for all these purposes.

Statistical analyses were done using SPSS for Windows version 11.5. Firstly, to test the homogeneity between the treatment groups, the Mann-Whitney's U or Student's t test (depending on the normality assumption) was applied for quantitative control variables and the chi-square or Fisher's exact test for the qualitative ones. Pharmacokinetic parameters were tested for normal distribution by the Shapiro-Wilk's test and for variance homogeneity by the Levene's test. To compare formulations the analysis of variance (ANOVA) and the estimation of confidence intervals (90%) of the ratio were used. Vital signs and laboratory variables were treated using paired analysis (Student's t test or Wilcoxon's test, depending on the normality assumption), taking into account Bonferrony's adjustment for multiple comparisons. Regarding pharmacodynamics, formulations were compared at each time point using the Mann-Whitney's U test and the overall characteristics (mean and maximum effects, and time to reach the latter (T(Rmax)) were evaluated by the Mann-Whitney's U test. Adverse reactions frequencies were compared between formulations using the Fisher's exact test.

## Results

Thirty-four patients were recruited at the seven hospitals where they regularly received hemodialysis treatment. Twenty-two of them were included at the National Institute of Nephrology, Havana, where the trial took place for them. The other twelve from "Gustavo Aldereguía Lima" Hospital, Cienfuegos were studied at this same site.

The demographic and baseline characteristics of the patients as well as their toxic habits and causes of renal failure are shown in Table 1. Age and weight were highly variable within each group. Overall, ages ranged from 19 to 75 years and weights from 40 to 87 Kg. Groups were balanced regarding sex and race. Thirty patients had specified the cause of renal failure, prevailing diabetes mellitus and hypertension. The hypothesis of homogeneity between the groups was accepted.

One patient was excluded from pharmacokinetic and pharmacodynamic analysis due to a severe adverse event (ischemic chest pain), 24 hours after the administration of Formulation B. This patient was only included in safety evaluations. The rest of the patients complied with the treatment and follow-up as previewed.

### Pharmacokinetic analysis

**Figure 1:**
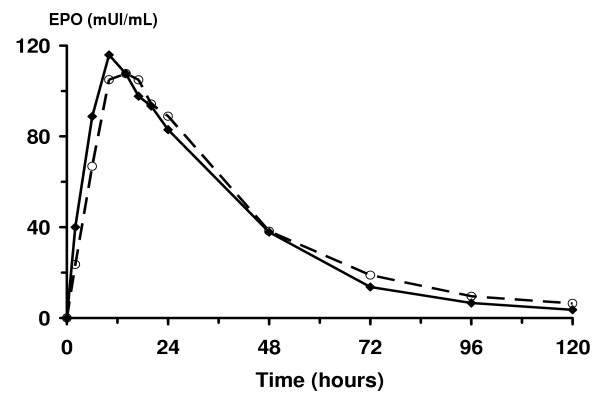
Average EPO concentration in serum. Data correspond to patients who received 100 IU/Kg of Heberitro (solid line) or Eprex (dashed line) at time 0. Standard deviations are not shown for the sake of simplicity of the illustration. Curves were not significantly different at any point in a multiple determinations ANOVA with Bonferrony's correction.

Except for one patient from each group all had small basal serum EPO levels (0.7 – 22 mIU/mL in group A and 0.9 – 34 mIU/mL in group B). After rHu EPO administration, some individual serum concentrations reached 200 mIU/mL. At 120 hours after the injection, serum EPO concentrations had returned to the initial values, so the AUC_120 _obtained covered, in all cases, more than 95% of the AUC extrapolated to infinite. The average concentration profiles obtained for both formulations were very similar (Figure 1).

Table 2 shows the results of the EPO pharmacokinetic comparisons. The differences between the means of any of the pharmacokinetic parameters (except Tmax) did not exceed the clinically significant level of 20%. The 90% confidence intervals were generally close to the 0.80 – 1.25 range. A formulation effect was not detected for any of the parameters.

### Pharmacodynamic analysis

**Figure 2:**
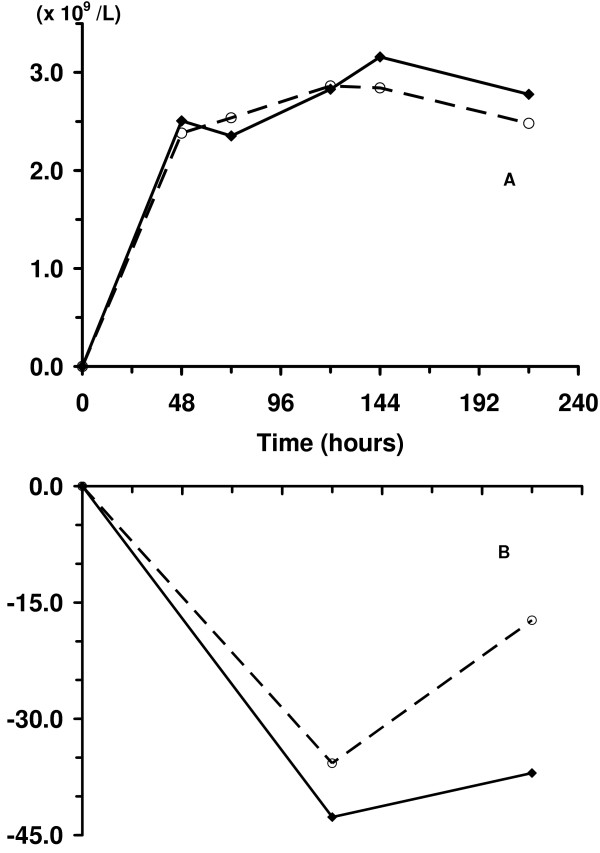
Pharmacodynamic variables. Data correspond to patients who received 100 IU/Kg of Heberitro (solid line) or Eprex (dashed line). (A): average reticulocyte counts increments; (B): percentage mean reduction in serum iron. Standard deviations are not shown for the sake of simplicity of the illustration.

Average reticulocyte count increments were very similar for both formulations, with the same profiles until 216 hours (Figure 2A). The larger increments were approximately 3 × 10^9 ^cells/L. The paired analysis yielded statistically significant reticulocyte count increments from 120 to 216 hours with respect to time 0. Both formulations behaved similarly (Table 3). The overall characteristics of the curves (maximum, means, and time to reach the maximum effect) did not show differences likewise (Table 4).

Figure 2B shows the percentage mean reduction in serum iron. A significant reduction was observed at 120 hours (paired analysis; Table 3). It was also comparable for both formulations (43 % for A and 36 % for B). A smaller percentage reduction appeared at 216 hours for formulation B. Quantitative analysis of the data also evidenced similar performance for both products (Table 4).

### Safety analysis

Twenty-three patients (67.6%) presented at least one adverse event during the study, 11 that received Formulation A (64.7%) and 12 Formulation B (70.6%). There were no differences between formulations concerning the adverse events, except for edemas, which were significantly more frequent with Formulation B (Table 5). The more frequent adverse events were pain at the site of injection (35.3%) and hypertension (29%). The events were mild to moderate and well controlled in general. Just one patient had a serious episode of angina 24 hours after Formulation B administration. This event was considered as "not related" with the product because the single dose applied does not justify an erythrocyte increment that could lead to this syndrome. Additionally, this patient had antecedents of ischemic cardiopathy. Differences in vital signs and other laboratory evaluations were not significant between formulations (data not shown). Hemoglobin and hematocrit were not affected with the single dose administered.

### Further follow-up

Further treatment with formulation A induced significant increases in hemoglobin and hematocrit (Table 6). All the patients, except 4 from both groups, were considered as responders since they increased at least the hematocrit in 5% and/or hemoglobin in 2 g/dL, after 3 months of treatment. During this period, the EPO-related adverse events were fever, chills and headache, each one recorded in one patient. No serum anti-EPO antibodies were detected in any patient.

## Discussion

This trial indicates that the compared formulations have similar pharmacokinetic and pharmacodynamic characteristics. The dose selected was adequate, since EPO titers in serum were easily detected and the treatment was quite well tolerated. Differences between formulations were not statistically significant for any of the calculated parameters. Regarding serum EPO profiles, differences were not statistically significant at any time point; the curves overlap. The fact that more than 80% of the AUC could be covered by the AUC_120 _obtained indicates that internal validity of the data is high. For pharmacokinetic parameters, except for CL, the 90%confidence intervals of the ratio between formulations were outside the 0.80 – 1.25 limits accepted for bioequivalence, but close to it. Taking into consideration that it was a parallel trial, using biotechnological products, in patients with anemia due to end-stage renal disease on hemodialysis and not in healthy subjects, a higher inter-individual variability could be expected. For example, patients' age range was quite wide. Then, a more flexible approach for bioequivalence criterion, at least 0.7 – 1.3, could be justified.

This variability occurred in the calculated pharmacokinetic parameters as well. Main parameters such as AUC, AUC_120 _or Cmax showed very small punctual differences (5% or less), but standard deviations, although similar between treatment groups, were large (coefficients of variation around 50%). Consequently, it was not possible that 90% confidence intervals fulfill with the 0.80 – 1.25 range.

This kind of study is usually done in healthy volunteers [12-15], since under a more controlled situation a smaller variability of the results is obtained. However, in this case investigators preferred to do it in the target patients since their renal failure condition would have considerable influence on EPO pharmacokinetics and pharmacodynamics. Besides, there was a risk due to EPO-dependent blood viscosity increase and hypertension [16], and Ethics Committees could have been reluctant to approve the protocol. In fact, there is very little safety information in the above cited reports on healthy volunteers.

On the other hand, for bioequivalence trials crossover treatment groups with an inter-treatment washout period are preferred. This leads to estimate and to reduce the individual variability as the main variation source; therefore, statistical analyses have more power. However, anemic patients with renal chronic failure need continuous EPO for their red cell synthesis. To submit them to a washout period implied to wait again for the irruption of anemia and this procedure was not ethically acceptable. After this trial, the patients continued EPO treatment under phase II trial conditions. They were monitored during this period and the results show that the study product was effective in increasing their hemoglobin and hematocrit values significantly.

In some situations, the intra and inter-subject variability can be equally or more important than the variation from an innovator product to a similar. In fact, the importance of the individual bioequivalence concept is recently being claimed, which would allow the scaling of the established intervals based on the individual comparison and the subject – formulation interaction [17,18].

For both formulations, Tmax was within the 12 – 18 hours reported for EPO, although a great variability is reported for this variable in renal patients [11,19-23]. Tmax is a discrete variable that depends on the experimental design so it cannot be interpreted as a continuous value and thus the small punctual difference observed looses practical significance. The European Guidelines recommend its analysis by non-parametric procedures [24]. Values for F, t_1/2 _and MRT were according to other studies, 18 – 49%, 15–25 hours and 25 – 45 hours, respectively [11,12,19,25-28]. Cmax, although continuous, depends much on the times at which samples are taken. Several reports propose to expand its bioequivalence range up to 30% [29-31], and in some cases this wider 90% CI can be accepted [24]. Cmax is considered either as a magnitude or as a rate of absorption parameter but does not accurately describe either process. Other parameters such MRT and CAV best describe absorption rates [32,33].

The results of pharmacodynamic variables (reticulocyte counts and serum iron) predict a possible therapeutic equivalence between the formulations, given their similitude. It is not possible to achieve significant increments in hematocrit or hemoglobin (main clinical endpoints) with a single EPO dose, but the reticulocyte count increase correlates with the clinical effect. Stable increments in hemoglobin and hematocrit are only reached after prolonged treatment, as was found in these patients. A multiple dose design is costly and complicated for this kind of trial. It is only justified for drugs with specific pharmacological characteristics (e.g. non-linear pharmacokinetics). Additionally, any eventual blood transfusion, which is often in these patients, would interfere with the results.

The pharmacodynamic curves were not well characterized by using pharmacokinetic-like parameters due to ethical reasons, since some patients needed to be transfused. Additionally, EPO receptor-binding dependent biological effects attain their maximum several days after administration, when EPO serum levels have returned to baseline [14,34]. Therefore the dissipation phases of the effects were not well covered.

The integration of pharmacokinetic and pharmacodynamic characterizations into drug development provides a scientific framework for its rational and efficient application [35,36], as has already been done for EPO in athletes [37]. Repeated administration of EPO to healthy subjects was more effective in stimulating a reticulocyte response than single-dose administration of the same total amount [14]. However, similar pharmacodynamic effects with dissimilar doses or schedules were also reported in healthy individuals [38].

Concerning safety, treatment was well tolerated, except for an episode of angina. The number of patients that presented adverse events was almost the same for both formulations. Many adverse events were observed during hemodialysis sessions or few hours after. Thus, it is very difficult to predict if those events were EPO- or dialysis-related. The most frequent event observed with recombinant EPO treatment is hypertension, but headache, flu-like syndrome, rash, vascular thrombosis and others can appear [2-4,16,39,40]. As a rule they appear after longer treatments, higher doses or due to a rapid hematocrit increase. During the follow-up period very few drug-related adverse events appeared, mostly mild.

The introduction of "biogenerics" to the market is a controversial matter nowadays. Two recombinant EPO molecules could be similar from the physicochemical point of view, but it is always possible that differences in producing strains and/or manufacturing processes turn out into small differences in composition (isoforms, host contaminants, ingredients, etc.) that could have clinical repercussion in terms of safety or efficacy. However the "process = product" dogma has been broken in the last years and the attitude of regulatory agencies is more towards accepting, on a case by case basis, the possibility of interchangeability of biological – derived products, mostly when they are highly purified and characterized molecules. In that sense, some examples exist already in the market such as different insulins, somatotrophins, and CHO cells-derived interferon beta from different manufacturing sites [41]. In fact, two EPREX formulations with different stabilizers were pharmacokinetically equivalent [42]. The different EPO preparations have been in the clinics for several years and substantial information concerning their safety and efficacy in their various indications has been gathered. It is thus unlikely that any unexpected general safety or efficacy issue can arise from the use of a well characterized EPO preparation.

The main concern has been the development of anti-EPO neutralizing antibodies and consequently, aplastic anemia [43]. This phenomenon has been related to packaging and storage conditions [44], but should be monitored carefully since it still appears, even at very low frequency. Canadian authors found very low frequency and concluded that a general screening is not justified, but only on resistant patients [45]. No serum anti-EPO antibodies were detected after 3 months of treatment in our study. However this is still a short period to derive any conclusion on the immunogenicity of the molecule.

The possibility that a home-made EPO reaches the market in a developing country is an attractive alternative in order to expand its use, given the high prices that the proprietary drugs have. The cost of EPO treatment is approximately 500 USD per month [46] in the United States using commercially available products. For example, in Cuba (11 million inhabitants) there are approximately 2000 renal patients in hemodialysis, but this figure should increase after an investment policy where new hemodialysis facilities are set up throughout the country. If EPO had to be imported to cover this population, the country should spend not less than 12 million USD per year for this purpose (not counting additional freight and commercial charges). To use the national product would permit a significant impact on health care, even if the "biogenerics" concept is not accepted. In the case of newly developed EPO preparations, the comparable pharmacokinetics and pharmacodynamics data can be the basis for its introduction in the national market. Then further efficacy information can be gathered as well as immunogenicity monitoring.

## Conclusion

Given that both formulations have similar pharmacokinetic, pharmacodynamic and short-term safety profiles, the newly developed product can be used in the clinics, with an appropriate efficacy, safety and immunogenicity monitoring.

## Competing interests

Authors IGG, PJPM, CMVS, HBL, and PALS are employees of the Center for Biological Research, which is part of the Center for Genetic Engineering and Biotechnology, Havana network, where human recombinant EPO is produced. The rest of the authors have no competing interests at all.

## Authors' contributions

JFPO and MCG and RHV took care of patient recruitment, management, and follow-up. IGG participated in the study design and coordination, EPO EIA determinations and wrote the manuscript draft. PJPM conceived the study and participated in the study design and coordination. CMVS participated in the study design and statistical analysis. THM and MLA carried out blood sampling and laboratory determinations. HLB developed the anti-EPO antibodies assay, its validation, and tested the patient's samples. YCK, YAA and AVB assisted as monitors. PALS took part in the design, results analysis and manuscript writing. All authors read and approved the final manuscript.

## Appendix

The other members of the Bioequivalence Study of Erythropoietin Group are as follows: Emilio Fors-López ("Carlos J. Finlay" Hospital, Havana), Rafael Cruz-Peinado ("Miguel Enríquez" Hospital, Havana), Diego Falcón ("Calixto García" Hospital, Havana), Eusebio González-Alvarez, Ortelio González-Flebes, Oscar E. Hernández-Díaz ("Aleyda Fernández Chardiet" Hospital, Güines), Isabel Herrera, Zenaida Hernández, Leonel Soto-León ("Abel Santamaría" Hospital, Pinar del Río), Hortensia Gautier-du Defaix, Delfina Pedroso-Comino, Tsunami Alvarez, Maiker Bello, Yanet Santos, (National Institute of Nephrology, Havana), Orelys Olivert-Hernández, Pedro Muñiz-Olite, Aldo González-Pérez, René Rosales-González, Carmen L. Rodríguez-Santiago, Francisca González ("Gustavo Aldereguía Lima" Hospital, Cienfuegos), Gisou Díaz-Rojo, Elizeth García-Iglesias, Giselle Pentón-Rol, Damián Mainet-González (Center for Biological Research, Havana), Pedro R. Casanova-Arias, Maité Reyes-Cañedo (Center for Genetic Engineering and Biotechnology, Havana), Roger Delgado-Figueroa, Karina Alfonso-Alfonso (National Center of Clinical Trials, Havana), Jorge Ducongé-Soler (University of Havana, Institute of Pharmacy and Food, Havana, Cuba).

## Pre-publication history

The pre-publication history for this paper can be accessed here:



## Figures and Tables

**Table 1:** Demographic and baseline characteristics of the patients

**Variables**		**Formulation A**	**Formulation B**	**Total**	**p**
**N**		**17**	**17**	**34**	

Age (years)		52 ± 15 (19–72)	52 ± 16 (23–75)	52 ± 15 (19–75)	0.90*
Weight (Kg)		60 ± 12 (40–87)	59 ± 13 (42–83)	59 ± 12 (40–87)	0.82*
Sex	Male	11 (64.7%)	8 (47.1%)	19 (55.9%)	0.30^Δ^
	Female	6 (35.3%)	9 (52.9%)	15 (44.1%)	
Race	White	8 (47.1%)	10 (58.8%)	18 (52.9%)	0.73^!^
	Non-white	9 (52.9%)	7 (41.2%)	16 (47.1%)	
Smoking		4 (23.5%)	1 (5.9%)	5 (14.7%)	0.33^!^
Alcohol consumption		4 (23.5%)	1 (5.9%)	5 (14.7%)	0.33^!^
Coffee drinking		7 (41.2%)	7 (41.2%)	14 (41.2%)	1.00^Δ^
Causes of renal failure					
Diabetes Mellitus		7 (46.7%)	6 (40.0%)	13 (43.3%)	0.71^Δ^
Hypertension		5 (33.3%)	4 (26.7%)	9 (30.0%)	1.00^!^
Other glomerulopathies		1 (6.7%)	1 (6.7%)	2 (6.7%)	1.00^!^
Cystic disease		1 (6.7%)	3 (20.0%)	4 (5.6%)	0.60^!^

Data are reported as mean ± standard deviation (range) or number of patients (%).

*: Student's t test; ^Δ^: chi-square test; !: Fisher's exact test

**Table 2:** Pharmacokinetic parameters calculated from the EPO concentration in serum

**Parameter**	**Formulation A N = 17**	**Formulation B N = 16**	**Difference (%)**	**p**	**90 % CI**
AUC_120_(mUI·h/mL)	4500 ± 2281	4654 ± 2221	5.1	0.49	(0.71 – 1.27)
AUC (mUI·h/mL)	4667 ± 2319	4918 ± 2113	3.3	0.66	(0.71 – 1.32)
Cmax (mUI/ml)	119.1 ± 56.4	119.7 ± 60.5	0.5	0.96	(0.73 – 1.35)
Tmax (h)	13.9 ± 9.9	18.1 ± 14.9	23.2	0.08	-
λ(h^-1^)	0.040 ± 0.018	0.037 ± 0.015	10.8	0.49	(0.89 – 1.32)
t_1/2 _(h)	20.0 ± 7.9	22.5 ± 12.1	11.1	0.29	(0.69 – 1.14)
CAV	0.027 ± 0.008	0.024 ± 0.007	8.0	0.38	(0.94 – 1.31)
F	0.29 ± 0.14	0.31 ± 0.14	6.4	0.53	(0.75 – 1.17)
MRT (h)	33.9 ± 10.7	42.3 ± 20.8	19.8	0.15	-
CL (mL/h·Kg)	6.3 ± 0.3	6.3 ± 0.4	0.5	0.55	(0.98 – 1.03)
Vd (mL/Kg)	182 ± 74	202 ± 107	10.0	0.67	(0.70 – 1.15)

Data are reported as mean ± standard deviation

CI: confidence intervals of the mean ratio; for Tmax and MRT, 90 %CI could not be estimated for a parallel design.

p: ANOVA between groups

**Table 3:** Reticulocyte Counts Increments and Percentage Reduction of Serum Iron during the study

	**Formulation A N= 17**	**Formulation B N= 16**	**p A vs. B**
**Reticulocyte counts**	**Increments (×10^9^/L) (p for paired test) significance level p = 0.010**		

T48 -T0	2.5 ± 4.2 (0.109)	2.4 ± 3.4 (0.028)	0.694
T72 -T0	2.4 ± 3.4 (0.078)	2.5 ± 3.7 (0.011)	0.520
T120 -T0	2.8 ± 2.9 (0.001)	2.9 ± 2.8 (0.002)	0.986
T144 -T0	3.2 ± 2.8 (<0.001)	2.8 ± 2.7 (0.002)	0.552
T216-T0	2.8 ± 2.2 (< 0.001)	2.5 ± 2.4 (0.001)	0.407
**Serum iron**	**% Reduction (p for paired test) significance level: p = 0.025**		

T120 -T0	42.7 ± 20.5 (0.001)	35.7 ± 17.1 (0.001)	0.205
T216-T0	37.0 ± 21.4 (0.050)	17.3 ± 19.4 (0.541)	0.805

Data are reported as mean ± standard deviation.

Paired tests vs. baseline were done by the Wilcoxon's test and comparison between formulations at each time by the Mann-Whitney's U test.

**Table 4:** Overall quantitative analysis of reticulocyte counts (×10^9^/L) and serum iron percentage reduction

**Analysis**	**Formulation A N= 17**	**Formulation B N= 16**	**p (Mann-Whitney's U test)**
**Reticulocyte counts (×10^9^/L)**			

Mean	2.7 ± 2.9	2.6 ± 2.7	0.678
Maximum	4.3 ± 3.9	3.9 ± 3.9	0.397
T(Rmax) (hours)	146.8 ± 59.9	130.5 ± 51.8	0.201
**Serum iron percentage reduction**			

Mean	39.8 ± 18.4	26.5 ± 13.1	0.265
Maximum	47.3 ± 19.2	39.2 ± 15.2	0.355
T(Rmax) (hours)	160.9 ± 61.6	148.5 ± 53.5	0.365

Data are reported as mean ± standard deviation.

These data correspond to the 34 patients who received one of the EPO formulations.

T(Rmax): Time to reach maximum response.

**Table 5:** Frequency of adverse events during the study

**Variable**		**Formulation A N = 17**	**Formulation B N = 17**	**p (Fisher's test)**
Adverse events	Yes	11 (64.7%)	12 (70.6%)	1.00
	No	6 (35.3%)	5 (29.4%)	
Specific adverse event	Chest pain	0	1 (5.9%)	0.50
	TGP increase	1 (5.9%)	0	0.50
	Tachycardia	1 (5.9%)	4 (23.5%)	0.17
	Headache	4 (23.5%)	3 (17.6%)	0.50
	Abdominal pain	0	1 (5.9%)	0.50
	Asthenia	1 (5.9%)	3 (17.6%)	0.30
	Diarrhea	0	2 (3.6%)	0.24
	Dyspnea	0	2 (3.6%)	0.24
	Somnolence	0	1 (1.8%)	0.50
	Neck pain	1 (5.9%)	0	0.50
	Hypertension	6 (35.3%)	4 (23.5%)	0.35
	Pain at site of injection	6 (35.3%)	6 (35.3%)	1.00
	Bone pain	0	1 (5.9%)	0.50
	Edema	0	6 (35.3%)	0.009*
	Fever	2 (11.8%)	5 (29.4%)	0.20
	Hypotension	5 (29.4%)	2 (11.8%)	0.20
	General malaise	1 (5.9%)	1 (5.9%)	0.50
	Pneumonia	0	1 (5.9%)	0.50
	Pruritus	1 (5.9%)	3 (17.6%)	0.30
	Chills	2 (11.8%)	2 (11.8%)	0.70
	Vomiting	3 (17.6%)	6 (35.3%)	0.22

Data are presented as number of individuals with each adverse reaction (%).

**Table 6:** Efficacy evaluation during further follow-up. Both groups of patients received formulation A, 30 UI per Kg 3 times per week, subcutaneously.

**Variable**	**Day**	**Group A**	**Group B**
**Hemoglobin (g/dL)**	0	8.8 ± 1.7	9.0 ± 1.4
	90	10.5 ± 1.4	11.4 ± 1.6
	p (90 vs. 0)	0.027	0.004

**Hematocrit (%)**	0	25.6 ± 4.4	26.9 ± 5.9
	90	35.5 ± 3.2	36.4 ± 5.1
	p (90 vs. 0)	< 0.001	0.001

Data are reported as mean ± standard deviation.

Paired tests vs. day 0 were done by paired t test.
